# Occupational Therapy in Severe Mental Disorder—A Self-Controlled Quasi-Experimental Study

**DOI:** 10.3390/healthcare10030493

**Published:** 2022-03-08

**Authors:** Efrén Valverde-Bolivar, Agustín Javier Simonelli-Muñoz, José Miguel Rivera-Caravaca, Juana Inés Gallego-Gómez, María Teresa Rodríguez González-Moro, José Joaquín García-Arenas

**Affiliations:** 1 Faculty of Health Sciences, Catholic University of Murcia, 30107 Murcia, Spain; erizofrenico@gmail.com (E.V.-B.); mtrodriguez@ucam.edu (M.T.R.G.-M.); jjgarcia2@ucam.edu (J.J.G.-A.); 2 Department of Nursing, Physiotherapy and Medicine, University of Almería, 04007 Almería, Spain; 3 Department of Cardiology, Instituto Murciano de Investigación Biosanitaria (IMIB-Arrixaca), CIBERCV, Hospital Clínico Universitario Virgen de la Arrixaca, University of Murcia, 30120 Murcia, Spain

**Keywords:** occupational therapy, activity, intervention, perception, severe mental disorder

## Abstract

Severe mental disorder (SMD) produces a significant functional limitation that affects the performance of daily activities. The occupational therapist intervenes on this limitation by seeking greater autonomy of these patients through specific activities. This study aims to identify the main limitations of people with SMD and to examine whether an occupational intervention has any effect in helping to overcome or ameliorate these limitations. A quasi-experimental study including 103 participants was carried out. An evaluation using the World Health Organization Disability Assessment Schedule (WHODAS 2.0) questionnaire was performed before and after the intervention. Within the activity program, those with a higher attendance rating during cognitive stimulation, cooking workshop, therapeutic walks, relaxation, and creative activities were mainly men. Both patients and professionals indicated that Understanding and Communicating, Participation in Society, and Activities of Daily Living were the main perceived limitations. Upon discharge, patients and professionals reported positive outcomes. The intervention programs carried out by occupational therapy, along with the other aspects of the treatment that SMD patients received, played an important part in improving the performance and occupational interests of these patients.

## 1. Introduction

During the last decades, a considerable effort has been made to improve therapeutic approaches in mental health. Severe Mental Disorder (SMD) [1] is one of the fields that has gained more attention. SMD encompasses a series of disorders such as schizophrenia, schizotypal personality disorder, delusional disorder, schizoaffective disorder, other specified schizophrenia spectrum or other psychotic disorders, bipolar I disorder with psychotic features, major depressive disorder with psychotic features, and obsessive-compulsive disorder [2]. Its diagnosis includes at least two of the following criteria [3]:

1. Unemployment (including sheltered or supported employment), severely limited skills, or poor work history.
2. Support to apply for public financial assistance to reintegrate into society.
3. Difficulties in establishing or maintaining personal social support systems.
4. Need for help with daily living activities, such as hygiene, food preparation, or financial management.
5. Inappropriate social behavior that determines psychiatric or judicial assistance.

These disorders, among others, produce a significant functional limitation in carrying out Activities of Daily Living (ADLs). Therefore, one of the main challenges in the intervention of individuals with SMD is to improve their ADLs. To meet individuals’ needs, interventions should be focused on a collaborative relationship between healthcare professionals, patients, and families/caregivers [4]. Thus, the therapeutic approach must be adapted to interdisciplinary teams, supported by pharmacological treatment and others. By adopting this perspective, patients are treated from an integrated and holistic point of view, considering the different human, biological and psychological dimensions in family and social life [5]. With this information in mind, occupational therapy has an essential role in the management of patients with mental disorders, especially in the prognosis [6,7]. The main aim is the search for greater autonomy and, in the field of mental health, the reintegration of the patient using the community as an occupational element, basing the interventions on the model of human occupation [8,9].

The objectives of the present study are to identify the principal limitations of people with SMD and to examine whether an occupational intervention has any effect in helping to overcome or improve these limitations.

## 2. Materials and Methods

### 2.1. Design of Study

This is a quasi-experimental, self-controlled analytical design with pre- and post-intervention measures. 

### 2.2. Sample

We included patients admitted to the Regional Unit of Medium Stay of the Region of Murcia (URME) from September 2020 to December 2021. This unit belongs to the psychiatric hospital Román Alberca (Murcia, Spain). All patients had SMD requiring an extended admission. The following inclusion criteria were considered: age ≥18 years, absence of active symptoms (delusions, hallucinations, psychomotor agitation, or other uncommon symptoms) that interfere with the dynamics, and being willing to participate in the different programs carried out by occupational therapists. 

First, the purpose of the study was explained to the participants, and the informed consent was required. Confidentiality of data was guaranteed at all times, respecting the agreements of the Declaration of Helsinki.

A sample size (n) of approximately 100 participants was sought to obtain the statistical significance required for this type of research [10,11,12]. The final sample consisted of 103 participants, of which 89 were able to be reevaluated within the study timeframe, whereas 14 patients were unable to be reevaluated at the end of the study since they were discharged. In addition, 2 patients refused to participate in the study and therefore were not included. 

### 2.3. Data Collection and Variables

The approach was assessed by occupational therapy using the gathering of data. Two evaluations were carried out after the sociodemographic analysis of the population of individuals; the first one upon admission of the patients and the other after the intervention, just before they were discharged. These evaluations were carried out both by the patients themselves and by the health professionals.

Initially¸ sociodemographic data that complement the questionnaire variables were recorded including sex, age, educational level, family situation, and the number of hospitalizations.

The research instrument for collecting information about the occupational performance was the World Health Organization Disability Assessment Schedule (WHODAS 2.0) questionnaire [13]. The WHODAS questionnaire is a research instrument that frequently provides information about disabilities in different populations, such as schizophrenia [14], and has been translated and validated in Spanish [15]. This tool consists of 7 factors that showed acceptable reliable indices in each of the measurements. Responses score in a qualitative five-point Likert-type scale from 1 (None) to 5 (Extreme). Higher scores indicate a more severe disability.

### 2.4. Intervention

Once admitted to the unit, all patients had a team of professionals formed by a psychiatrist, a psychologist, a social worker, an occupational therapist, a nurse and a nursing assistant. The way the Unit works was explained to all users during this first stage. 

At two weeks of admission, an initial assessment was made to record the specific needs of each one, to draw up the Individual Recovery Plan (IRP) and to specify the occupational objectives, which were agreed with the user. 

The program of activities proposed was aimed at training deficiencies detected in the users in order to promote their autonomy, and three packages of activities were established. The first package, common to all participants, included working on activities of daily living (basic and instrumental). The second package of activities was aimed at improving communication and interaction among users. Finally, the third package included tasks to work on social participation in the community. These last two packages of activities were of voluntary participation, so that regardless of the initial planning, the users had the possibility of carrying out other activities offered, depending on the individual’s needs. A record of participation was kept. We were able to observe, among other aspects, that the patients with the greatest number of admissions were those who had the greatest difficulty in getting involved in the activities, tending to remain inactive for long periods of time.

Specifically, the procedures focused on the development and acquisition of competencies in activities of daily living such as:

i. Basic Activities of Daily Living (BADL).
ii. Eating: 18 users required support to follow regular eating schedules and healthier eating habits.
iii. Bathing: We worked with 12 users in order to establish a bathing routine.
iv. Dressing: 9 users required indications for changing clothes or adapting them according to weather conditions.
v. Hygiene: 56 users needed intervention to establish adequate hand and dental hygiene.
vi. Instrumental Activities of Daily Living (IADLs)
vii. Use of public transportation: 4 users were trained to acquire the necessary autonomy.
viii. Money management: 12 users were intervened to plan expenses and income in order to adapt to their economy.
ix. Personal shopping: 54 users required follow-up to make purchases in the community.
x. Autonomy in the home (Cooking): 67 users worked on the preparation of recipes, including tasks such as the purchase of ingredients, use of household appliances, cleaning of utensils, application of different cooking techniques.
xi. Laundry care: 14 users were trained in the use of the washing machine and dryer.
xii. Use of new technologies; 37 users needed help in the use of the computer, cell phone or tablet.
xiii. Assessment of the home; 7 users were visited to check whether their home met the requirements for habitability.

Regarding the package of activities that were voluntary, the most demanded were cognitive stimulation activities, relaxation, therapeutic outings, cooking group and creative workshop. On the other hand, the activities with the least participation were cineforum, vegetable garden and swimming. In addition, there were other activities in which other professionals from the multidisciplinary team (psychiatrists, psychologists and nurses) were responsible, such as education for families, psychoeducation or health education. No record of participation in these groups was kept, but we are aware that their implementation had an influence on the patient’s improvement.

Additionally, the participants evaluated the professionals through a satisfaction survey shortly before their discharge.

### 2.5. Data Analysis and Treatment

The data was processed by SPSS version 27.0 (SPSS, Inc., Chicago, IL, USA), using Student’s *t*-test comparing two variables, parametric multi-variant analysis ANOVA and linear correlations. All data obtained from the multivariate analysis are expressed as M ± SD o t ± DT.

First, descriptive parameters for user participation in occupational interventions were calculated. Secondly, several analyses of mean differences were conducted in order to check the variations of the factors of the WHODAS between admission and discharge of the patient and between self-evaluation of the patient and evaluation of the professional (taking into account the staff’s patient evaluation). Finally, the differences in means were analyzed, taking into account some of the user’s sociodemographic characteristics, which are included at the beginning of the results since they include parameters from both groups of the other means. Subsequently, the Statistics of the demographic variables previously collected were calculated in order to be analyzed.

## 3. Results

We included 103 patients, 49.8% women with a mean age of 41.4 ± 10.2 years. Regarding the level of education, 5.8% had not received any education, 46.6% had completed primary school, 35.9% had secondary education, and 11.7% had a university title. In terms of their familial situation, 25.2% lived alone while 74.8% lived with someone. Concerning the indication for admission, 50% were admitted by physician’s indication/judicial authorization, 47% at their own request and 3% by judicial order. The main mental disorder were (F20–29) Schizophrenia, schizotypal disorders and delusional disorders (69%); (F60–69) Personality and behavioral disorders in adults (14%); (F30–39) Mood [affective] disorders (10%), (F10–19) Mental and behavioral disorders due to the use of psychotropic substances (5%), and the remaining 2% were (F40–48) Neurotic disorders, stress-related disorders and somatoform disorders. The pharmacological treatment received by patients is individualized and adapted to the clinical manifestations of the disease they are suffering from and to their general state of health. Specifically, according to diagnosis, the most commonly used treatments were: neuroleptics/antipsychotics, as the medication of choice in schizophrenias and other psychotic disorders (61 patients). These were received in various forms, some of the most commonly used are long-acting injectables (from 15 days to 3 months), mainly for those patients who have difficulty in maintaining adherence to treatment (25 patients). In patients with mood disorders, mood stabilizers or eutimizers such as lithium (1 patient) and valproic acid (2 patients) and antidepressants in cases of dysthymia (5 patients) were used. In personality disorders, a combination of drugs was usually applied, but in these cases it was shown that the best treatment is psychotherapy (12 patients). Anxiolytics (benzodiazepines) and hypnotics or sleep inducers were used in most of the disorders, being the most used in the Unit because most of the patients (80 patients) presented problems to control anxiety and sleep problems.

From the overall cohort, 89 (86.4%) participants completed the reassessment (i.e., had a pre- and post-test evaluation); whereas the remaining 14 (13.6%) only completed the initial assessment. The reasons why the post-test was not performed were: voluntary discharge (6 patients), escape from the unit and subsequent administrative discharge (4 patients), refusal to be re-evaluated (3 patients), and alteration of the psychopathological state at the time of discharge (1 patient). Mean duration of admission was 119 ± 56.07 days. After discharge, 81% of the users were referred to their mental health center.

An analysis was carried out to check the difference in factors relating to the WHODAS before and after the intervention according to the patient’s self-assessment. As shown in Table 1, significant differences were found in all factors. Specifically, the self-evaluation in each of the factors was more positive (lower) at discharged than when they were admitted, showing an improvement in the patients’ self-perception regarding their limitations. The factors in which the change was greater were Understanding and Communicating and Participation in Society, together with Activities of Daily Living.

The same analyses were carried out according the professional’s perspective. As shown in Table 2, there were significant differences in all the WHODAS factors. Self-evaluations by the patients and the professionals’ assessments found a more positive outcome at discharged compared to admission. The analysis showed improvements for both patients and professionals. Regarding the patients’ self-evaluation, the factors in which there was a more significant change were Understanding and Communicating and Participation in Society. These two factors were also those that received the most negative evaluation from the professionals, together with Activities of Daily Living and Interpersonal Relationships. In contrast to the patients’ view, Interpersonal Relationships were not recognized as a significant barrier.

A repeated measures analysis was also performed with an intra-subject factor (Time: Admission vs. Discharge) and a factor between subjects (Evaluator: Patient vs. Expert) in order to check whether there were differences between the patient’s self-assessment and the professional’s assessment, as well as the time when the measure was taken. The results only showed a significant interaction in Interpersonal Relationships (IR) (Table 3). Specifically, the evaluation of patient in the IR was lower at discharge than at the beginning of the interventions. However, when he patients acted as evaluators, patients’ assessment was lower compared to professionals’ assessment. No significant differences were found in other factors, so both the patients’ self-evaluation and the professionals’ evaluation were similar at admission and at discharge.

Finally, different analyses were carried out to check whether there were differences in the different WHODAS factors, taking into account some sociodemographic variables and, in other cases, whether there was a correlation. The results presented are mainly related to sex, age, educational level, familial situation, and the number of previous admissions. We only observed significant differences in the WHODAS evaluations due to the large number of analyses for each factor.

In the case of sex, differences in the PS factor were found in the self-assessment of the patients upon admission (*t* = 3.060, *p* = 0.003). Specifically, women (2.70 ± 0.81) presented a higher self-assessment than men (2.26 ± 0.66). On the other hand, in the self-evaluation they carried out when they were discharged, differences were found in the factors CM (*t* = 2.408, *p* = 0.020), PC (*t* = 2.026, *p* = 0.049) and PS (*t* = 2.176, *p* = 0.032). In the three cases, women (CM: 1.53 ± 0.80; PC: 1.37 ± 0.67; PS: 1.97 ± 0.54) presented higher self-evaluations than men (CM: 1.18 ± 0.42; PC: 1.13 ± 0.24; PS: 1.73 ± 0.49). 

Regarding the professionals’ evaluations, in reference to the admission of the patients, differences were found in factors CM (*t* = 2.520, *p* = 0.014), ADLs (*t* = 4.187, *p* < 0.001) and PS (*t* = 2.872, *p* = 0.005). Again, women (CM: 1.71 ± 0.81; PS: M = 2.73, DT = 0.72) obtained higher evaluations than men (CM: 1.36 ± 0.47; PS: 2.36 ± 0.50) in CM and PS; however, men (3.07 ± 0.58) scored higher than women (2.36 ± 0.99) in ADLs. Regarding discharge, differences were found in CM (*t* = 2.402, *p* = 0.020) and ADLs (*t* = 2.052, *p* = 0.044). In CM, women (1.48 ± 0.71) scored higher than men (1.17 ± 0.40) and in ADLs, men (2.13 ± 0.72) scored higher than women (1.77 ± 0.88).

In the case of age, in the self-assessment of patients upon admission, only a negative correlation was found between age and UC (*r* = −0.246, *p* = 0.012), while in the self-assessment carried out when they were discharged, no correlation was found. When the evaluation was carried out by professionals, age was positively correlated with CM (*r* = 0.307, *p* = 0.002) and PC (*r* = 0.240, *p* = 0.014) during admission, while at discharge only a positive relationship was found with PC (*r* = 0.304, *p* = 0.003).

In the case of the number of previous admissions to the hospital, correlations were also made. Upon admission, patients were given a self-assessment showing a significant positive relationship with UC (*r* = 0.202, *p* = 0.04), whereas no significant correlation was found at discharge. During the professional’s evaluation, the same positive relationship between the number of previous admissions and UC was significant (*r* = 0.203, *p* = 0.039), and again at discharge, no significant relationship was found.

In the case of education, the patients’ self-evaluations found no significant difference (*p* = 0.094) neither at discharge nor at admission. However, in the professional’s evaluation, significant differences were found. At admission, significant differences appeared in UC (*F* = 3.120, *p* = 0.029). A post-hoc analysis by the Tukey method revealed that the differences were found between those with primary education (2.62 ± 0.59) and university education (2.06 ± 0.67), the former being the ones with the most difficulties. At discharge, significant differences were found in UC (*F* = 3.404, *p* = 0.021) and ADLs (*F* = 2.758, *p* = 0.047). In the case of UC, those with primary education had a worse prognosis (M = 1.93, SD = 0.56) than those with secondary education (1.60 ± 0.48) or university education (1.46 ± 0.40); while in the case of ADLs, who had primary education (2.21 ± 0.75) had a worse prognosis than those with secondary education (*M* = 1.71 ± 0.75). 

Regarding the familial situation, significant differences were revealed in CM during the self-evaluation at admission (*t* = 2.708, *p* = 0.008). Those who lived with a family (1.64 ± 0.85) had a worse prognosis than those living alone (1.32 ± 0.34). No significant differences were shown at discharge. On the other hand, regarding the evaluation of the professionals, no significant differences were found either at admission or discharge.

Lastly regarding the personal satisfaction survey, 92.20% of the users stated that they had received “good or very good treatment” and 76.60% considered that “the therapy helped them a lot”.

## 4. Discussion

The objectives of the present study were to identify the main limitations of people with SMD and to investigate whether an occupational intervention has any effect in helping to overcome or ameliorate these limitations. Different patients with SMD were evaluated after their admission to the Unit and just before discharge to measure their limitations. These evaluations included a study of their sociodemographic parameters, which is the core of this research. A biased is possible with self-assessments by the patients due to their social cognition [16]. Therefore, these evaluations were carried out both by patients and by professionals. 

During their admission, patients participated in several interventions carried out through occupational therapy, intended to improve the evaluations of their occupational performance. The results showed that both the patients and the professionals indicated that Understanding and Communicating, Participation in Society, and Activities of Daily Living were the main limitations they encountered [17]. However, only professionals indicated Interpersonal Relationships as a barrier. Most of the participants were not in the early stages of the disease, which does not significantly affect their level of functionality despite the pharmacological treatment prescribed to each subject [18]. Schennach et al., in their study on the evolution of patients with schizophrenia 2 years after hospital discharge, observed that despite the natural and unstable course of the disease 64% presented satisfactory daily functioning [19].

On the other hand, the evaluation was more positive after the participation in occupational tasks. The improvement was especially significant in Understanding and Communicating and in Participation in Society. When comparing the evaluations made by patients and professionals, differences were only found in Interpersonal Relationships. This result shows that the patients overestimated their abilities to relate to other people. One possibility is that the patients were unaware of their illness symptoms. In this regard, when conducting the evaluations, it was observed that certain patients were unable to perceive their current situation when they were admitted. However, more time spent in the unit, together with psychopharmacological treatment and establishing a socio-occupational routine [20], allowed the patients to acquire a greater awareness of their situation. Therefore, the individuals may have acquired a capacity of insight [21], allowing them to obtain awareness of their current situation [22]. On the contrary, those patients whose admission is prolonged have more difficulty in establishing an occupational balance, remaining inactive for long periods of time, thus fostering negative aspects of the disease itself, such as anhedonia or abulia.

Nevertheless, no significant differences were found in the other factors concluding that the patients’ self-evaluation was similar to the professionals’ evaluation. Therefore, no evidence was found that the evolution of the disease and the associated deterioration at cognitive level [23] were affecting psychosocial functioning [24]. Moreover, self-evaluations indicated that patients had acquired awareness of their illness.

Finally, some differences were also found according to sociodemographic characteristics. Based on sex, women had lower self-assessment scores, especially in Community Mobility, Personal Care, and Participation in Society. However, the professionals only evaluated women lower in Community Mobility and men in Activities of Daily Living. Age was associated with a lower rating in personal care. Likewise, an increased number of admissions was related to a more negative evaluation in Understanding and Communicating. In contrast, those who had lower levels of education, presented a lower Understanding and Communicating and were not capable of carrying out Activities of Daily Living. Pathology by itself was not shown to be a determining effect on the beneficial outcome of therapy, in which the family environment or the socioeconomic situation of the patient may influence as associated factors. These aspects should also be analyzed in future research. On the other hand, as in other studies [25], those who lived with their family had better ratings in Community Mobility, although it was only perceived by patients.

In summary, SMD causes a significant disability [26] in those who suffer from it. This disease is associated with functional problems, including the social and occupational functioning [27]. Therefore, adherence to a socio-occupational routine for people with SMD is complex, and a possible explanation is based on the functional impairment of such a diverse population [28].

There are few studies focused on the occupational interests of people with SMD identifying the main occupational limitations of these patients [29]. In the present study, in addition to following the Model of Human Occupation [30], we considered the environment [31], and cultural variables during activities since these factors could influence the performance of each activity.

## 5. Limitations

There are some limitations to be considered. As this is a quasi-experimental study, it would be necessary to develop another investigation with an experimental and a control group. Further research with a larger sample would be necessary.

## 6. Conclusions

Intervention programs conducted by occupational therapy together with psychopharmacological treatment have assisted in improving performance and occupational interests in patients with SMD. This improvement is perceived by patients and professionals alike after the treatment in the Medium Stay Unit.

## Figures and Tables

**Table 1:** Simple effects of time on the different factors of the World Health Organization Disability Assessment Schedule (WHODAS) according to the patient’s self-evaluation.

	Admission M ^a^ (SD ^b^)	Discharge M ^a^ (SD ^b^)	*t*	*p*	Cohen’s d
UC ^c^	2.25 (0.80)	1.60 (0.61)	6.943	0.000	1.07
CM ^d^	1.56 (0.78)	1.32 (0.62)	3.427	0.001	0.34
PC ^e^	1.39 (0.68)	1.23 (0.47)	2.355	0.021	0.27
IR ^f^	2.16 (0.80)	1.79 (0.70)	4.023	0.000	0.49
ADLs ^g^	2.24 (1.06)	1.63 (0.82)	5.077	0.000	0.64
PS ^h^	2.42 (0.72)	1.83 (0.52)	2.742	0.023	0.94

^a^ M: Mean; ^b^ SD: Standard Deviation; t: Student’s *t*; *p*: Statistical Significance; ^c^ UC; Understanding and Communicating; ^d^ CM; Community Mobility; ^e^ PC; Personal Care; ^f^ IR: Interpersonal Relationships; ^g^ ADLs: Activities of Daily Living; ^h^ PS; Participation in Society.

**Table 2:** Simple effects of time on the different WHODAS factors according to the professional’s evaluation.

	Admission M ^a^ (SD ^b^)	Discharge M^a^ (SD ^b^)	*t*	*p*	Cohen’s d
UC ^c^	2.47 (0.64)	1.77 (0.56)	12.277	0.000	1.16
CM ^d^	1.50 (0.67)	1.29 (0.56)	3.170	0.002	0.34
PC ^e^	1.60 (0.63)	1.34 (0.49)	5.095	0.000	0.46
IR ^f^	2.56 (0.69)	2.03 (0.60)	8.102	0.000	0.82
ADLs ^g^	2.78 (0.87)	1.99 (0.80)	9.300	0.000	0.95
PS ^h^	2.50 (0.62)	1.87 (0.47)	8.812	0.000	1.15

^a^ M: Mean; ^b^ SD: Standard Deviation; t: Student’s *t*; *p*: Statistical Significance; ^c^ UC; Understanding and Communicating; ^d^ CM; Community Mobility; ^e^ PC; Personal Care; ^f^ IR: Interpersonal Relationships; ^g^ ADLs: Activities of Daily Living; ^h^ PS; Participation in Society.

**Table 3:** Interaction between time and the evaluator in the different factors of the WHODAS.

	Patient		Professional				
	Admission M ^a^ (SD ^b^)	Discharge M ^a^ (SD ^b^)	Admission M ^a^ (SD ^b^)	Discharge M ^a^ (SD ^b^)	F	*p*	η^2^_p_
UC ^c^	2.25 (0.80)	1.60 (0.61)	2.47 (0.64)	1.77 (0.56)	0.759	0.386	0.008
CM ^d^	1.56 (0.78)	1.32 (0.62)	1.50 (0.67)	1.29 (0.56)	0.344	0.559	0.004
PC ^e^	1.39 (0.68)	1.23 (0.47)	1.60 (0.63)	1.34 (0.49)	2.683	0.105	0.029
IR ^f^	2.16 (0.80)	1.79 (0.70)	2.56 (0.69)	2.03 (0.60)	5.313	0.023	0.056
ADLs ^g^	2.24 (1.06)	1.63 (0.82)	2.78 (0.87)	1.99 (0.80)	3.080	0.083	0.033
PS ^h^	2.42 (0.72)	1.83 (0.52)	2.50 (0.62)	1.87 (0.47)	1.159	0.285	0.013

^a^ M: Mean; ^b^ SD: Standard Deviation; t: Student’s *t*; p: Statistical Significance; ^c^ UC; Understanding and Communicating; ^d^ CM; Community Mobility; ^e^ PC; Personal Care; ^f^ IR: Interpersonal Relationships; ^g^ ADLs: Activities of Daily Living; ^h^ PS; Participation in Society.

## Data Availability

The data presented in this study are available on request from the corresponding author.
